# Self‐Harm–Associated Gastrointestinal Foreign Bodies: National Patterns of Management and Outcomes

**DOI:** 10.1111/den.70256

**Published:** 2026-08-07

**Authors:** David Wong Arias, Spencer R. Goble, Mauricio Torrealba

**Affiliations:** ^1^ Department of Medicine Hennepin Healthcare Minneapolis Minnesota USA; ^2^ Division of Gastroenterology, Hepatology, and Nutrition University of Minnesota Minneapolis Minnesota USA; ^3^ Department of Gastroenterology and Hepatology Hennepin Healthcare Minneapolis Minnesota USA

**Keywords:** endoscopy, foreign bodies, gastrointestinal, gastrointestinal tract, operative, self‐injurious behavior, surgical procedures

## Abstract

**Objectives:**

Gastrointestinal foreign body ingestion or insertion can be an act of intentional self‐harm. We aimed to provide a large‐scale assessment of this clinical scenario.

**Methods:**

This is a retrospective cross‐sectional analysis of adult patients who presented with gastrointestinal foreign bodies and intent to self‐harm. Hospitalizations were identified using the National Inpatient Sample from 2016 to 2023. Each hospitalization was characterized by the management methods used including no intervention, endoscopy, and surgery. Evaluated outcomes included ICU‐level care, length of stay, and cost. Additionally, we assessed factors associated with the need for surgery after endoscopy.

**Results:**

A total of 16,150 hospitalizations were identified. The mean age was 33.9 years and most patients were male (65.7%). Over one‐third (35.2%) of patients had a cluster B personality disorder while 39.3% had schizophrenia and 24.2% were incarcerated. The majority (61.2%) of patients were managed with endoscopy alone while 26.4% required no intervention and 12.3% required surgery. ICU‐level care was increased in those who required surgery, but the difference was not statistically significant (4.5% vs. 3.0%; OR 1.54, 95% CI: 0.91–2.62, *p* = 0.111). Small bowel location was associated with the need for surgery after endoscopy (aOR 2.50, 95% CI 1.91–3.28; *p* < 0.001). Over 12,000 hospital days and $25,000,000 were attributable to self‐harm‐associated gastrointestinal foreign bodies annually.

**Discussion:**

Self‐harm–associated gastrointestinal foreign bodies occur predominantly in patients with significant psychiatric comorbidity. Although mortality and severe morbidity are uncommon, healthcare utilization is substantial. Small bowel location is a key predictor of the need for surgical intervention.

## 1 Introduction

Gastrointestinal (GI) foreign bodies are a common clinical problem encountered by gastroenterologists. Unlike pediatric cases, adult GI foreign bodies frequently occur in the setting of psychiatric illness, substance use, or incarceration [1, 2, 3]. In some adults, ingestion or insertion of a foreign body represents an act of self‐harm, which may reflect suicidal intent or serve as a means of secondary gain, such as temporary removal from custodial settings [1, 4, 5, 6]. Patients may intentionally select objects with a high risk of injury, including razor blades, glass, or other sharp materials [1, 5, 7]. Recurrent episodes are common in this population, and several single‐center retrospective studies and case reports have demonstrated substantial healthcare utilization and ongoing management challenges associated with repeated presentations [1, 5, 7, 8].

Management strategies for GI foreign bodies include observation, endoscopic removal, and surgical intervention [9, 10, 11]. Evidence‐based guidelines provide recommendations to guide management decisions based on object characteristics, location, and clinical presentation [9, 10]. In most cases, endoscopy represents the preferred initial therapeutic approach, with surgery generally reserved for failed endoscopic retrieval, objects beyond endoscopic reach, or complications such as obstruction, perforation, or sepsis [9, 10]. While procedural approaches to foreign body management are well described, outcomes among patients with self‐harm–associated GI foreign bodies—and factors associated with escalation of care—remain poorly characterized using large‐scale, generalizable data.

To address these gaps, we performed a retrospective analysis using the National Inpatient Sample (NIS). By providing nationally representative data, this study aims to better define characteristics and outcomes in this complex patient population.

## 2 Methods

### 2.1 Study Design and Database Description

This is a retrospective, cross‐sectional analysis of self‐harm–associated GI foreign body hospitalizations. Hospitalizations from 2016 through 2023 were identified using the NIS. The NIS is an all‐payer United States inpatient database developed as part of the Healthcare Cost and Utilization Project (HCUP). It is designed to approximate the entire United States inpatient population and includes approximately seven million hospitalizations annually. Hospitalizations are selected using a systematic, stratified sampling design. HCUP provides standardized weighting procedures that, when applied to the NIS, yield nationally representative estimates. These standardized weights were applied in all analyses, and all results are presented as weighted national estimates. The NIS is a deidentified, publicly available database, and Institutional Review Board approval was waived.

### 2.2 Study Sample and Variables

All adult hospitalizations from 2016 through 2023 with a primary diagnosis of a GI foreign body and a secondary diagnosis indicating self‐harm intention were included. Diagnoses were defined using relevant ICD‐10 diagnostic codes (Table S1). In the NIS, the primary diagnosis represents the condition chiefly responsible for the admission. We restricted the cohort to hospitalizations with a primary diagnosis of GI foreign body to ensure inclusion of patients who presented with a foreign body and to exclude patients who ingested or inserted a foreign body during hospitalization for an unrelated condition.

Demographic, clinical, and hospital‐level characteristics were collected for each hospitalization, with assessed variables summarized in Table 1. Foreign body location was determined based on the primary diagnostic code. All procedures were identified using ICD‐10 procedure codes. To determine whether patients underwent endoscopic and/or surgical intervention, all ICD‐10 procedure codes associated with each hospitalization were reviewed. Procedure codes classified as endoscopic procedures or foreign body–related surgical interventions are listed in Table S2, along with their weighted frequencies.

**TABLE 1:** Characteristics of hospitalizations for gastrointestinal foreign bodies in patients with self‐harm intention.

Age, years % (*n* = 16,150)	
18–30	42.7
31–49	48.8
50+	8.5
Female, % (*n* = 16,140)	34.3
Race, % (*n* = 13,735)	
White	61.2
Black	20.8
Hispanic	12.7
Asian or pacific islander	1.1
Other	4.2
United States hospital region, % (*n* = 14,015)	
Northeast	16.6
Midwest	21.5
South	35.6
West	26.3
Teaching hospital, % (*n* = 16,150)	84.5
Charlson Comorbidity Index, % (*n* = 16,150)	
0	58.9
1	29.3
2	8.0
3+	3.9
Major depressive disorder, % (*n* = 16,150)	35.1
Generalized anxiety disorder, % (*n* = 16,150)	31.0
Post‐traumatic stress disorder, % (*n* = 16,150)	26.7
Bipolar disorder, % (*n* = 16,150)	34.9
Schizophrenia, % (*n* = 16,150)	39.3
Cluster B personality disorder, % (*n* = 16,150)	35.2
Alcohol use, % (*n* = 16,150)	8.8
Incarceration, % (*n* = 16,150)	24.2
Concurrent genitourinary foreign body, % (*n* = 16,150)	2.6
Sharp object, % (*n* = 16,150)	21.1
Object location, % (*n* = 16,150)	
Esophagus	6.6
Stomach	43.7
Small bowel	15.0
Colon	13.8
Rectum or anus	3.6
Not specified	17.4

### 2.3 Study Objectives

The primary objective of this study was to describe the prevalence of self‐harm–associated GI foreign body hospitalizations and to describe the patient population with an emphasis on psychiatric comorbidities. Secondary outcomes included assessing outcomes across different management strategies and identifying factors associated with the need for surgical intervention.

Management strategies were categorized as follows: no intervention (no documented endoscopy or surgery), endoscopy only, endoscopy followed by surgery, surgery only, and surgery followed by endoscopy. Hospitalizations were classified as endoscopy followed by surgery if surgical intervention was documented on the same hospital day or after an endoscopic procedure. This approach was based on established clinical guidelines and practice patterns that emphasize an endoscopy‐first strategy when feasible, with surgery typically reserved for unsuccessful endoscopic management or complications. Hospitalizations were classified as surgery followed by endoscopy only if endoscopy was documented at least one calendar day after surgery. Notably, the NIS does not specify procedure indication, so we are unable to determine exactly why endoscopy was required after surgery in relevant patients.

Outcomes compared across management strategies included intensive care unit (ICU)–level care, GI resection, sepsis, length of stay (LOS), and hospitalization cost in United States dollars. ICU‐level care was defined as the presence of any of the following: positive pressure ventilation for greater than 24 consecutive hours, mechanical ventilation for greater than 24 consecutive hours, central venous catheter placement, vasopressor use, or cardiopulmonary resuscitation. A separate analysis excluding central venous catheter placement is included in the supporting information. Sepsis was identified using relevant ICD‐10 diagnostic codes. GI resection was defined by ICD‐10 procedure codes.

To assess factors associated with surgical intervention, the following variables were evaluated: age, sex, Charlson Comorbidity Index, presence of mood or psychotic disorders, presence of a cluster B personality disorder, incarceration status, sharp object characteristics, and foreign body location.

### 2.4 Statistical Analysis

Patient and hospital characteristics were summarized using weighted proportions. When comparing outcomes across management strategies, categorical variables were reported as weighted proportions, and continuous variables were reported as medians with interquartile ranges to account for the right‐skewed distributions of LOS and cost. Survey‐weighted medians and interquartile ranges were calculated using probability weights.

*p*‐values for comparisons between management strategies were derived using Rao–Scott adjusted *χ* ^2^ tests for categorical variables and design‐based F tests for continuous variables, accounting for the complex survey design of the NIS.

Multivariable survey‐weighted logistic regression was used to identify factors independently associated with surgical intervention. Covariates were selected a priori based on clinical plausibility and existing literature, rather than univariable statistical significance [9, 12]. Variables likely to represent downstream complications of management (e.g., sepsis, perforation, or ICU‐level care) were not included in the primary model. Clinically relevant associations are presented in the main text and figures, while univariable results and results from the full multivariable model are provided in the supporting information (Tables S3–S6). All analyses were performed using Stata/BE version 17.0 (StataCorp, College Station, TX).

## 3 Results

### 3.1 Sociodemographic and Clinical Characteristics

A total of 16,150 self‐harm–associated GI foreign body hospitalizations were identified. The mean age of the cohort was 33.9 years, with over 90% of patients younger than 50 years (Table 1). The cohort was predominantly male (65.7%) and white (61.2%). Overall comorbidity burden was low, with a mean Charlson Comorbidity Index of 0.6, and 58.9% of patients having a score of zero. Psychiatric disorders were common; schizophrenia was the most frequently documented diagnosis, present in 39.3% of hospitalizations. Nearly one‐quarter of patients (24.2%) were incarcerated and incarcerated patients were more likely to be male and present with sharp objects (Tables S7–S11). Foreign body location was specified in 82.6% of hospitalizations, with over half of objects (50.3%) located in the esophagus or stomach.

### 3.2 Comparison of Outcomes by Management Pathway

**FIGURE 1:**
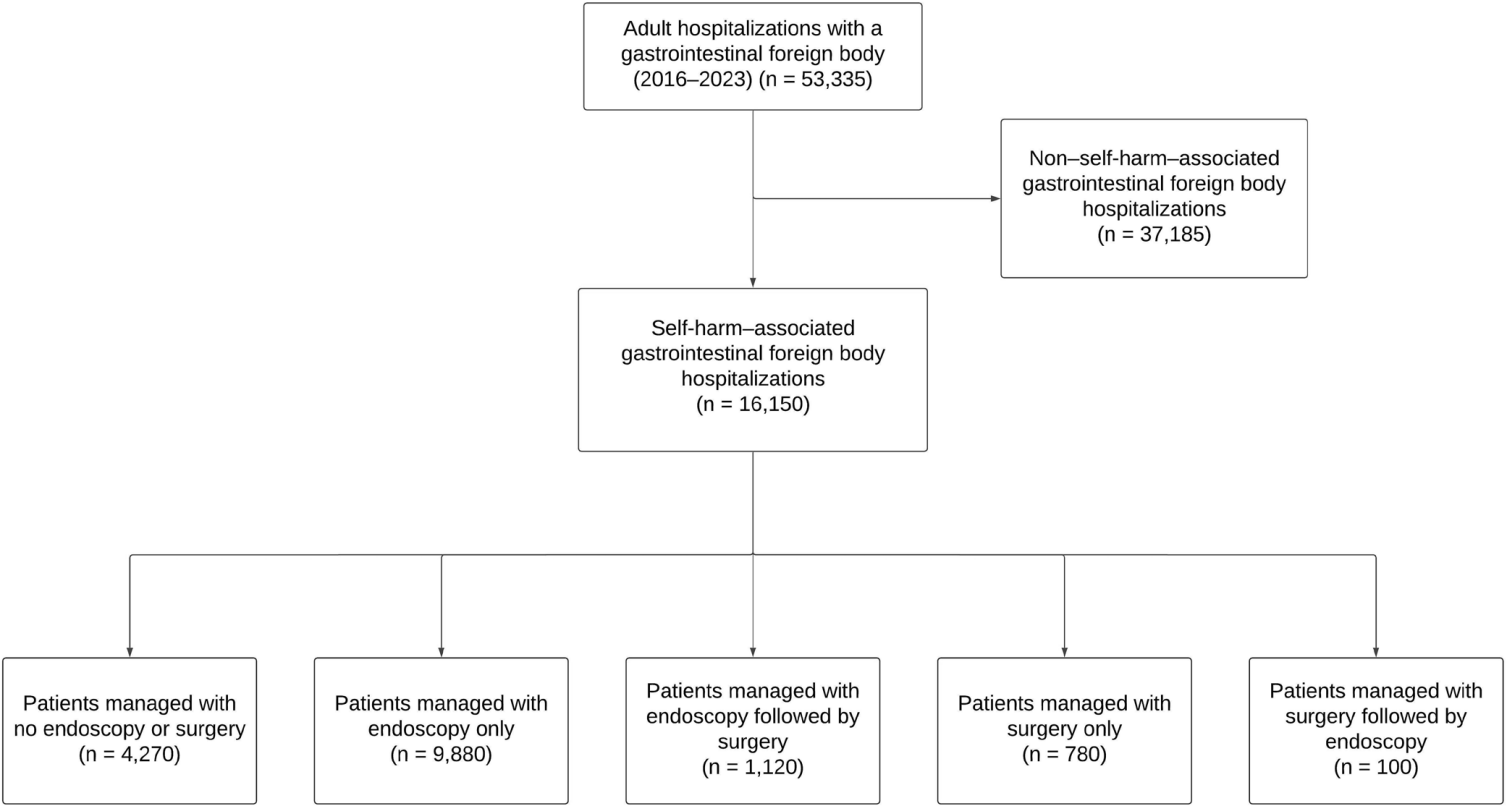
Derivation of a cohort of hospitalizations for self‐harm–associated gastrointestinal foreign bodies using the National Inpatient Sample.

The most common management strategy was endoscopy only (61.2%), followed by no intervention (26.4%), endoscopy followed by surgery (6.9%), surgery only (4.8%), and surgery followed by endoscopy (0.6%) (Figure 1). Overall mortality was rare, with only five deaths recorded (0.3 per 1000 hospitalizations). ICU‐level care was required in 510 hospitalizations (3.2%). The highest proportion of ICU‐level care occurred among patients managed with surgery followed by endoscopy (10.0%), whereas the lowest proportion occurred in the no‐intervention group (0.4%) (Table 2) (Table S12). ICU‐level care was more frequent among patients who underwent any surgical intervention compared with those managed without surgery, although this difference did not reach statistical significance (4.5% vs. 3.0%; OR 1.54, 95% CI 0.91–2.62; *p* = 0.111).

**TABLE 2:** Clinical outcomes by management pathway among patients hospitalized with self‐harm–associated gastrointestinal foreign bodies.

Outcome	No intervention (*n* = 4270)	Endoscopy only (*n* = 9880)	Endoscopy followed by surgery (*n* = 1120)	Surgery only (*n* = 780)	Surgery followed by endoscopy (*n* = 100)	*p*
ICU‐level care	15 (0.4%)	405 (4.1%)	60 (5.4%)	20 (2.6%)	10 (10.0%)	< 0.001
Gastrointestinal resection	—	—	240 (21.4%)	210 (26.9%)	35 (35.0%)	< 0.001
Sepsis	10 (0.2%)	100 (1.0%)	95 (8.5%)	25 (3.2%)	5 (5.0%)	< 0.001
Length of stay, median (IQR)	3 (2–5)	4 (2–7)	7 (4–12)	7 (4–11)	6 (4–17)	< 0.001
Cost, median USD (IQR)	5715 (3671‐8673)	9157 (5922‐14,308)	19,475 (13,327‐31,641)	16,874 (10,662‐26,357)	24,228 (10,124‐41,941)	< 0.001

*Note:* Values are presented as weighted *n* (%) for categorical variables and weighted median (interquartile range) for continuous variables. *p*‐values reflect design‐based *F* tests for continuous variables and Rao–Scott adjusted *χ* ^2^ tests for categorical variables, accounting for the complex survey design of the National Inpatient Sample.

GI resection was documented in 485 hospitalizations, accounting for 24.3% of hospitalizations involving any surgical intervention. Sepsis occurred in 235 hospitalizations (1.5%) and was substantially more common among patients requiring surgery compared with those managed without surgery (6.3% vs. 0.8%; OR 8.51, 95% CI 4.78–15.15; *p* < 0.001).

Across the eight‐year study period, 98,195 hospitalization days (approximately 12,274 days per year) were attributable to self‐harm–associated GI foreign bodies. The median LOS for the overall cohort was 4 days (IQR 2–7 days) and was longer among patients who underwent surgery (median 7 days, IQR 4–12 days) compared with those managed without surgery (median 3 days, IQR 2–6 days). The total cost of hospitalizations was $205,972,094 (approximately $25,746,511 per year). The median cost per hospitalization was $8690 (IQR $5418–$14,661) and was highest among patients managed with surgery followed by endoscopy (median $24,228).

### 3.3 Object Location and Management Pathway

Among hospitalizations with a specified foreign body location (*n* = 11,345), most objects were located in the stomach (62.2%), followed by the small bowel (21.3%), colon (19.6%), esophagus (9.3%), and rectum or anus (5.1%). Surgical intervention was most common for small bowel foreign bodies (23.6%), followed by those in the colon (13.0%), rectum or anus (12.9%), stomach (10.9%), and esophagus (9.0%). The proportion of hospitalizations requiring no intervention ranged from 10.4% for esophageal foreign bodies to 35.5% for colonic foreign bodies (Table 3) (Table S11). Endoscopy was performed in 68.7% of cases overall, with the highest utilization for esophageal foreign bodies (88.7%), followed by stomach (80.9%), rectal or anal (77.6%), colonic (59.8%), and small bowel (52.1%) foreign bodies.

**TABLE 3:** Management pathways stratified by foreign body location among patients hospitalized with self‐harm–associated gastrointestinal foreign bodies.

Foreign body location	No intervention (*n* = 2980)	Endoscopy only (*n* = 8565)	Endoscopy followed by surgery (*n* = 1025)	Surgery only (*n* = 675)	Surgery followed by endoscopy (*n* = 100)
Esophagus (*n* = 1060)	110 (10.4%)	855 (80.7%)	80 (7.6%)	10 (0.9%)	5 (0.5%)
Stomach (*n* = 7060)	1120 (15.9%)	5170 (73.2%)	500 (7.1%)	230 (3.3%)	40 (0.6%)
Small bowel (*n* = 2420)	855 (35.3%)	995 (41.1%)	235 (9.7%)	305 (12.6%)	30 (1.2%)
Colon (*n* = 2225)	790 (35.5%)	1145 (51.5%)	165 (7.4%)	105 (4.7%)	20 (0.9%)
Rectum or anus (*n* = 580)	105 (18.1%)	400 (69.0%)	45 (7.8%)	25 (4.3%)	5 (0.9%)

*Note: p*‐value = 0.004. *p*‐value reflects a design‐based *F* test from a survey‐weighted linear regression model assessing the association between foreign body location and management pathway, accounting for the complex survey design of the National Inpatient Sample.

### 3.4 Variables Associated With Surgical Intervention

**FIGURE 2:**
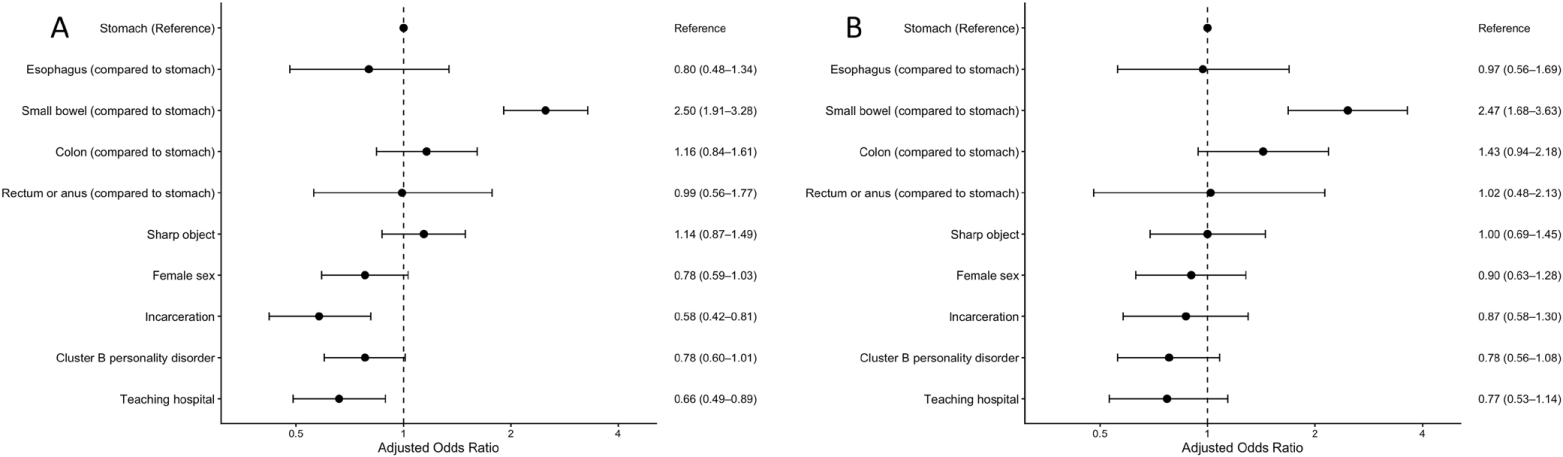
Multivariable predictors of surgical intervention and endoscopic failure in self‐harm–associated gastrointestinal foreign bodies. Forest plots displaying adjusted odds ratios (aORs) with 95% confidence intervals for (A) need for surgical intervention among all hospitalizations and (B) need for subsequent surgery following an initial endoscopic attempt. Endoscopic failure was defined as surgical intervention after endoscopy.

Compared with patients with gastric foreign bodies, those with small bowel foreign bodies had significantly higher odds of requiring surgical intervention (aOR 2.50, 95% CI 1.91–3.28; *p* < 0.001) (Figure 2). No other assessed variable was associated with a statistically significant increase in surgical intervention. The need for surgery did not significantly differ by year (Table S14). Surgery was more frequently utilized in patients presenting with sharp foreign bodies, although this difference was not statistically significant (14.1% vs. 11.9%; aOR 1.14, 95% CI 0.87–1.49; *p* = 0.330). Incarcerated patients and those treated at teaching hospitals were less likely to undergo surgery (10.2% vs. 13.1% and 11.6% vs. 16.5%, respectively), and these associations remained significant after multivariable adjustment (Figure 2).

### 3.5 Variables Associated With Surgical Intervention After Endoscopy

Among patients who underwent endoscopy without prior surgery (*n* = 11,000), subsequent surgical intervention was required in 10.2%. Rates ranged from 8.6% for esophageal foreign bodies to 19.1% for small bowel foreign bodies. On multivariable analysis, small bowel location (compared with stomach) was independently associated with an increased likelihood of subsequent surgical intervention (19.1% vs. 8.8%; aOR 2.47, 95% CI 1.68–3.63; *p* < 0.001). No other assessed variable was significantly associated with subsequent surgical management (Figure 2).

## 4 Discussion

In this nationally representative cohort of more than 16,000 hospitalizations for self‐harm–associated GI foreign bodies, several important patterns emerged. Admissions primarily involved young adults with a high prevalence of psychiatric disease and incarceration. Endoscopy was the predominant management strategy. Surgical intervention was required in a minority of cases but was associated with longer hospitalization, higher cost, and greater morbidity. Small bowel location emerged as the strongest independent predictor of surgical intervention.

Psychiatric disorders were highly prevalent, with schizophrenia documented in nearly 40% of hospitalizations, and mood and personality disorders also frequently observed. These findings are consistent with prior reports describing strong associations between serious mental illness and intentional foreign body ingestion or insertion [13, 14, 15, 16]. Prior studies have demonstrated frequent recurrent ingestion or insertion events in this population, and because the NIS captures hospitalizations rather than individual patients, it is likely that some patients contributed multiple admissions [4, 5, 15, 16, 17, 18].

Although psychiatric and personality disorders were common in the cohort, they did not appear to be associated with worse outcomes such as the need for surgical intervention. We feel that while psychiatric comorbidities may contribute to object ingestion or insertion, other factors such as timing of presentation, object characteristics, and object location are more likely to influence management and outcomes. However, certainly the management of psychiatric and personality disorder comorbidities should be addressed to attempt to prevent future episodes. The NIS does not include information on whether psychiatry was consulted or patient follow‐up information. Prior studies have suggested several interventions, such as dialectical behavioral therapy, cognitive behavioral therapy, anti‐depressants, and anti‐psychotics, may be beneficial in addressing underlying mental health conditions in patients with recurrent foreign body presentations [4, 5, 16]. However, high quality follow‐up data assessing the impact of these interventions is lacking and further work is needed to determine their efficacies.

Recurrent foreign body presentations have been described in incarcerated individuals [19, 20]. Incarceration rates were high in our cohort and interestingly, surgical rates were decreased in incarcerated individuals despite increased rates of sharp objects. The NIS does not have the granularity to determine exactly why surgical rates in incarcerated individuals are lower, but we hypothesize that it is due to earlier recognition of ingestion events within supervised correctional settings leading to more rapid medical evaluation. Differences in age, sex, and comorbidity burden also could explain this finding.

Only five deaths were recorded, and ICU‐level care was required in just 3.2% of hospitalizations. These findings suggest that while intentional GI foreign body presentations rarely result in death, they nonetheless represent a substantial burden to healthcare systems. Across the study period, these admissions accounted for more than 12,000 hospital days and approximately $25 million annually in inpatient costs. Prior single‐center studies have similarly demonstrated disproportionate healthcare utilization related to recurrent foreign body presentations and the logistical challenges posed by urgent or after‐hours endoscopic intervention, emphasizing the importance of preventing recurrent presentations [4, 7].

Surgical intervention was associated with increased rates of sepsis, prolonged hospitalization, and higher cost, likely reflecting greater underlying disease severity such as obstruction, perforation, or failed endoscopic retrieval, all of which represent potential indications for surgery [9, 10, 21, 22, 23]. Although ICU‐level care occurred more frequently among surgical cases, this difference did not remain statistically significant after adjustment, suggesting that even patients requiring operative management generally experience favorable outcomes when appropriately treated.

Foreign body location significantly influenced management strategy. Esophageal and gastric foreign bodies were most frequently managed endoscopically, consistent with accessibility via standard upper endoscopy. In contrast, small bowel foreign bodies were independently associated with increased odds of surgical intervention and need for surgery after attempted endoscopic extraction. This finding is clinically intuitive, as large portions of the small intestine remain beyond the reach of conventional endoscopy, and distal objects may present with obstruction or other complications necessitating operative management [24, 25, 26]. Similar associations between distal intestinal location and surgical need have been reported in prior studies [8, 10, 24, 27]. Together, these findings suggest that anatomic location represents a key determinant of escalation of care and may help inform early risk stratification.

Current American and European clinical guidelines suggest urgent endoscopy for reachable small bowel foreign objects that are sharp or larger (five to six centimeters) [9, 10]. Our findings emphasize the potential harm of small intestinal foreign bodies and suggest that prompt removal if objects are still reachable may improve outcomes, although more granular data on precise object location and endoscopy details are needed to inform future guidelines. Interestingly, teaching hospital status was associated with decreased surgical rates. It is possible that this is due to interinstitutional differences in the availability of balloon‐assisted enteroscopy which could allow for retrieval of more distal small bowel objects.

This study has limitations inherent to administrative database analyses. Self‐harm intent may be underreported, particularly in institutions lacking psychiatric resources, which could lead to selection bias. Object characteristics, timing of presentation, and local expertise are all clearly important aspects in the management of foreign bodies, and the NIS unfortunately does not have this granular level data. Additionally, the NIS captures hospitalizations, not individual patients. We suspect that numerous patients likely had recurrent presentations and it is possible that a small subset of patients with severe psychiatric disorders or incarceration accounted for a large proportion of hospitalizations. Our findings should be interpreted as hospitalization‐level findings and not descriptive for individual patients. Additionally, the NIS does not include patients who were not admitted, so patients discharged from the emergency department after object removal are not included. The NIS does not specify cause of death.

In conclusion, self‐harm–associated GI foreign body hospitalizations predominantly involve young adults with significant psychiatric comorbidity and frequently include incarcerated individuals. Endoscopy is utilized in most cases and is associated with low mortality and generally favorable outcomes. Surgical intervention, while necessary in selected patients, is associated with increased resource utilization and morbidity. Small bowel location represents the strongest predictor of the need for surgery. These findings provide nationally representative data describing real‐world management patterns and outcomes.

## Funding

The authors have nothing to report.

## Ethics Statement

The authors have nothing to report.

## Conflicts of Interest

The authors declare no conflicts of interest.

## Supporting information


**Table S1:** ICD‐10 diagnostic and procedural codes used to assess hospitalizations for self‐harm‐associated gastrointestinal foreign bodies.
**Table S2:** ICD‐10 procedure codes used to identify endoscopy and surgery in patients with self‐harm associated gastrointestinal foreign bodies.
**Table S3:** Univariable regression analysis assessing factors associated with the need for surgical intervention among patients hospitalized with self‐harm–associated gastrointestinal foreign bodies.
**Table S4:** Univariable regression analysis assessing factors associated with the need for surgical intervention after initial endoscopic attempt among patients hospitalized with self‐harm–associated gastrointestinal foreign bodies.
**Table S5:** Multivariable regression analysis assessing factors associated with the need for surgical intervention among patients hospitalized with self‐harm–associated gastrointestinal foreign bodies.
**Table S6:** Multivariable regression analysis assessing factors associated with the need for surgical intervention after initial endoscopic attempt among patients hospitalized with self‐harm–associated gastrointestinal foreign bodies.
**Table S7:** Characteristics of hospitalizations for gastrointestinal foreign bodies in patients with self‐harm intention, stratified by incarceration status.
**Table S8:** Multivariable logistic regression assessing factors associated with the need for surgical intervention among non‐incarcerated patients hospitalized with self‐harm–associated gastrointestinal foreign bodies.
**Table S9:** Multivariable logistic regression assessing factors associated with the need for surgical intervention after initial endoscopic attempt among non‐incarcerated patients hospitalized with self‐harm–associated gastrointestinal foreign bodies.
**Table S10:** Multivariable logistic regression assessing factors associated with the need for surgical intervention among incarcerated patients hospitalized with self‐harm–associated gastrointestinal foreign bodies.
**Table S11:** Multivariable logistic regression assessing factors associated with the need for surgical intervention after initial endoscopic attempt among incarcerated patients hospitalized with self‐harm–associated gastrointestinal foreign bodies.
**Table S12:** Need for ICU‐level care by management pathway among patients hospitalized with self‐harm–associated gastrointestinal foreign bodies.
**Table S13:** Multivariable logistic regression assessing factors associated with no intervention being performed among patients hospitalized with self‐harm–associated gastrointestinal foreign bodies.
**Table S14:** Need for surgical intervention and surgical intervention after endoscopy among patients hospitalized with self‐harm–associated gastrointestinal foreign bodies, stratified by year.

## Data Availability

The data that support the findings of this study are available in National Inpatient Sample at https://hcup‐us.ahrq.gov/nisoverview.jsp. These data were derived from the following resources available in the public domain: National Inpatient Sample, https://hcup‐us.ahrq.gov/nisoverview.jsp.
