# Supplementary material for: Self‐Harm–Associated Gastrointestinal Foreign Bodies: National Patterns of Management and Outcomes
Source: Dig Endosc. 2026 Aug 7;38(8):e70256. doi: 10.1111/den.70256 (PMC13449619; doi:10.1111/den.70256)
Supplement: Supplementary file 1 [file DEN-38-0-s001.docx]

**Self-Harm–Associated Gastrointestinal Foreign Bodies: National Patterns of Management and Outcomes**

Authors: David Wong Arias MD, Spencer R Goble MD, Mauricio Torrealba MD

Table of Contents:

Supplementary methods: ………………………………………………………………………….	2

Supplementary results: ………………………………………………………………………………………………………….. 14

Table S1. ICD-10 diagnostic and procedural codes used to assess hospitalizations for self-harm-associated gastrointestinal foreign bodies

| Diagnosis or procedure | ICD-10 Codes |
| --- | --- |
| Alcohol use | F10*, K700, K7010, K7030, K709 |
| Bipolar disorder | F31* |
| Cardiopulmonary resuscitation | 5A12012 |
| Central line placement | 06HN33Z, 06HM33Z, 05H633Z, 05H533Z, 05HN33Z, 05HM33Z, 05HM3DZ |
| Cluster B personality disorder | F602*, F603*, F604*, F6081* |
| Colonic foreign body | T184* |
| Continuous positive pressure ventilation | 5A094*, 5A095* |
| Esophageal foreign body | T1810*, T18198 |
| Generalized anxiety disorder | F41* |
| Genitourinary foreign body | T19* |
| Gastrointestinal resection | 0DB80ZZ, 0DTJ0ZZ, 0DB60ZZ, 0DT80ZZ, 0DBH0ZZ, 0DTF0ZZ, 0DTH0ZZ, 0DBB0ZZ, 0DTJ4ZZ, 0DBN8ZZ, 0DBL0ZZ, 0DBN0ZZ, 0DBU0ZZ, 0DBA0ZZ, 0DB84ZZ, 0DBF0ZZ, 0DBM0ZZ, 0DB30ZZ, 0DBB4ZZ, 0DBU0ZX, 0DTL0ZZ, 0DB60Z3, 0DB20ZZ, 0DTB0ZZ, 0DBE0ZZ, 0DB64ZZ, 0DB40ZZ, 0DB64ZX, 0DTN0ZZ, 0DBE8ZX, 0DB80ZX, 0DB10ZZ |
| Incarceration | Z651, Y92149 |
| Major depressive disorder | F32*, F33* |
| Mechanical ventilation | 5A1935Z, 5A1945Z |
| Post-traumatic stress disorder | F431* |
| Rectum or anus foreign body | T185* |
| Schizophrenia | F200, F201, F202, F203, F205, F2081, F2089, F209, F22, F23, F250, F251, F258, F259, F28, F29 |
| Sepsis | T814*, A40*, A41* |
| Self-harm intention | T1491*, X7*, X80-84*, Z915*, R45851, R4588* |
| Sharp object | T188*, T189* |
| Small bowel foreign body | T183 |
| Stomach foreign body | T182* |
| Vasopressor use | 3E043XZ, 3E033XZ |

Table S2. ICD-10 procedure codes used to identify endoscopy and surgery in patients with self-harm associated gastrointestinal foreign bodies

| Intervention | **PCS** | **Definition** | Weighted Count |
| --- | --- | --- | --- |
| Endoscopy | 0DC68ZZ | Extirpation of Matter from Stomach, Via Natural or Artificial Opening Endoscopic | 5705 |
| Endoscopy | 0DJ08ZZ | Inspection of Upper Intestinal Tract, Via Natural or Artificial Opening Endoscopic | 3565 |
| Endoscopy | 0DC98ZZ | Extirpation of Matter from Duodenum, Via Natural or Artificial Opening Endoscopic | 1150 |
| Endoscopy | 0DJD8ZZ | Inspection of Lower Intestinal Tract, Via Natural or Artificial Opening Endoscopic | 835 |
| Endoscopy | 0DCH8ZZ | Extirpation of Matter from Cecum, Via Natural or Artificial Opening Endoscopic | 705 |
| Surgery | 0DC60ZZ | Extirpation of Matter from Stomach, Open Approach | 570 |
| Endoscopy | 0DC58ZZ | Extirpation of Matter from Esophagus, Via Natural or Artificial Opening Endoscopic | 360 |
| Endoscopy | 0DCP8ZZ | Extirpation of Matter from Rectum, Via Natural or Artificial Opening Endoscopic | 355 |
| Endoscopy | 0DCK8ZZ | Extirpation of Matter from Ascending Colon, Via Natural or Artificial Opening Endoscopic | 355 |
| Endoscopy | 0DB68ZX | Excision of Stomach, Via Natural or Artificial Opening Endoscopic, Diagnostic | 355 |
| Endoscopy | 0DC78ZZ | Extirpation of Matter from Stomach, Pylorus, Via Natural or Artificial Opening Endoscopic | 320 |
| Endoscopy | 0DC18ZZ | Extirpation of Matter from Upper Esophagus, Via Natural or Artificial Opening Endoscopic | 295 |
| Surgery | 0DC80ZZ | Extirpation of Matter from Small Intestine, Open Approach | 285 |
| Endoscopy | 0DC28ZZ | Extirpation of Matter from Middle Esophagus, Via Natural or Artificial Opening Endoscopic | 250 |
| Surgery | 0DN80ZZ | Release Small Intestine, Open Approach | 240 |
| Endoscopy | 0DC38ZZ | Extirpation of Matter from Lower Esophagus, Via Natural or Artificial Opening Endoscopic | 220 |
| Endoscopy | 0DCL8ZZ | Extirpation of Matter from Transverse Colon, Via Natural or Artificial Opening Endoscopic | 210 |
| Surgery | 0DNW0ZZ | Release Peritoneum, Open Approach | 185 |
| Endoscopy | 0DCN8ZZ | Extirpation of Matter from Sigmoid Colon, Via Natural or Artificial Opening Endoscopic | 185 |
| Endoscopy | 0DCA8ZZ | Extirpation of Matter from Jejunum, Via Natural or Artificial Opening Endoscopic | 180 |
| Surgery | 0DCA0ZZ | Extirpation of Matter from Jejunum, Open Approach | 160 |
| Endoscopy | 0DCP7ZZ | Extirpation of Matter from Rectum, Via Natural or Artificial Opening | 155 |
| Endoscopy | 0DB78ZX | Excision of Stomach, Pylorus, Via Natural or Artificial Opening Endoscopic, Diagnostic | 125 |
| Surgery | 0DCH0ZZ | Extirpation of Matter from Cecum, Open Approach | 115 |
| Surgery | 0DB80ZZ | Excision of Small Intestine, Open Approach | 110 |
| Surgery | 0DQ80ZZ | Repair Small Intestine, Open Approach | 110 |
| Surgery | 0WQF0ZZ | Repair Abdominal Wall, Open Approach | 105 |
| Endoscopy | 0W3P8ZZ | Control Bleeding in Gastrointestinal Tract, Via Natural or Artificial Opening Endoscopic | 105 |
| Surgery | 0DC90ZZ | Extirpation of Matter from Duodenum, Open Approach | 100 |
| Endoscopy | 0DC48ZZ | Extirpation of Matter from Esophagogastric Junction, Via Natural or Artificial Opening Endoscopic | 95 |
| Surgery | 0DCL0ZZ | Extirpation of Matter from Transverse Colon, Open Approach | 85 |
| Surgery | 0DTJ0ZZ | Resection of Appendix, Open Approach | 75 |
| Surgery | 0WJP0ZZ | Inspection of Gastrointestinal Tract, Open Approach | 75 |
| Endoscopy | 0DCM8ZZ | Extirpation of Matter from Descending Colon, Via Natural or Artificial Opening Endoscopic | 75 |
| Surgery | 0WCG0ZZ | Extirpation of Matter from Peritoneal Cavity, Open Approach | 70 |
| Surgery | 0DNU0ZZ | Release Omentum, Open Approach | 70 |
| Endoscopy | 0DC88ZZ | Extirpation of Matter from Small Intestine, Via Natural or Artificial Opening Endoscopic | 70 |
| Surgery | 0DB60ZZ | Excision of Stomach, Open Approach | 65 |
| Endoscopy | 0D9670Z | Drainage of Stomach with Drainage Device, Via Natural or Artificial Opening | 65 |
| Surgery | 0DCB0ZZ | Extirpation of Matter from Ileum, Open Approach | 60 |
| Surgery | 0DC64ZZ | Extirpation of Matter from Stomach, Percutaneous Endoscopic Approach | 60 |
| Surgery | 0JC80ZZ | Extirpation of Matter from Abdomen Subcutaneous Tissue and Fascia, Open Approach | 55 |
| Surgery | 0DQL0ZZ | Repair Transverse Colon, Open Approach | 55 |
| Endoscopy | 0DB98ZX | Excision of Duodenum, Via Natural or Artificial Opening Endoscopic, Diagnostic | 55 |
| Surgery | 0DNE0ZZ | Release Large Intestine, Open Approach | 50 |
| Endoscopy | 0DBP8ZX | Excision of Rectum, Via Natural or Artificial Opening Endoscopic, Diagnostic | 50 |
| Endoscopy | 0DJ68ZZ | Inspection of Stomach, Via Natural or Artificial Opening Endoscopic | 50 |
| Surgery | 0DCK0ZZ | Extirpation of Matter from Ascending Colon, Open Approach | 45 |
| Surgery | 0DJ00ZZ | Inspection of Upper Intestinal Tract, Open Approach | 45 |
| Endoscopy | 0DCE8ZZ | Extirpation of Matter from Large Intestine, Via Natural or Artificial Opening Endoscopic | 45 |
| Endoscopy | 0DCF8ZZ | Extirpation of Matter from Right Large Intestine, Via Natural or Artificial Opening Endoscopic | 45 |
| Endoscopy | 0DB58ZX | Excision of Esophagus, Via Natural or Artificial Opening Endoscopic, Diagnostic | 45 |
| Surgery | 0DCU0ZZ | Extirpation of Matter from Omentum, Open Approach | 40 |
| Surgery | 0DQ60ZZ | Repair Stomach, Open Approach | 40 |
| Surgery | 0WJG4ZZ | Inspection of Peritoneal Cavity, Percutaneous Endoscopic Approach | 35 |
| Surgery | 0DJD0ZZ | Inspection of Lower Intestinal Tract, Open Approach | 35 |
| Endoscopy | 0WJP8ZZ | Inspection of Gastrointestinal Tract, Via Natural or Artificial Opening Endoscopic Approach | 35 |
| Surgery | 0DT80ZZ | Resection of Small Intestine, Open Approach | 30 |
| Surgery | 0DBH0ZZ | Excision of Cecum, Open Approach | 30 |
| Surgery | 0DCN0ZZ | Extirpation of Matter from Sigmoid Colon, Open Approach | 30 |
| Surgery | 0DQ90ZZ | Repair Duodenum, Open Approach | 30 |
| Surgery | 0DNL0ZZ | Release Transverse Colon, Open Approach | 30 |
| Surgery | 0DJD4ZZ | Inspection of Lower Intestinal Tract, Percutaneous Endoscopic Approach | 30 |
| Surgery | 0WJG0ZZ | Inspection of Peritoneal Cavity, Open Approach | 30 |
| Surgery | 0DN84ZZ | Release Small Intestine, Percutaneous Endoscopic Approach | 30 |
| Endoscopy | 0DCC8ZZ | Extirpation of Matter from Ileocecal Valve, Via Natural or Artificial Opening Endoscopic | 30 |
| Endoscopy | 0DB38ZX | Excision of Lower Esophagus, Via Natural or Artificial Opening Endoscopic, Diagnostic | 30 |
| Surgery | 0DTF0ZZ | Resection of Right Large Intestine, Open Approach | 25 |
| Surgery | 0DTH0ZZ | Resection of Cecum, Open Approach | 25 |
| Surgery | 0DBB0ZZ | Excision of Ileum, Open Approach | 25 |
| Surgery | 0DTJ4ZZ | Resection of Appendix, Percutaneous Endoscopic Approach | 25 |
| Surgery | 0DC70ZZ | Extirpation of Matter from Stomach, Pylorus, Open Approach | 25 |
| Surgery | 0WCP0ZZ | Extirpation of Matter from Gastrointestinal Tract, Open Approach | 25 |
| Surgery | 0WCH0ZZ | Extirpation of Matter from Retroperitoneum, Open Approach | 25 |
| Surgery | 0DN60ZZ | Release Stomach, Open Approach | 25 |
| Surgery | 0DU607Z | Supplement Stomach with Autologous Tissue Substitute, Open Approach | 25 |
| Endoscopy | 0DCQ8ZZ | Extirpation of Matter from Anus, Via Natural or Artificial Opening Endoscopic | 25 |
| Surgery | 0DBN8ZZ | Excision of Sigmoid Colon, Via Natural or Artificial Opening Endoscopic | 20 |
| Surgery | 0DCA4ZZ | Extirpation of Matter from Jejunum, Percutaneous Endoscopic Approach | 20 |
| Surgery | 0DCP0ZZ | Extirpation of Matter from Rectum, Open Approach | 20 |
| Surgery | 0DCE0ZZ | Extirpation of Matter from Large Intestine, Open Approach | 20 |
| Surgery | 0DCV0ZZ | Extirpation of Matter from Mesentery, Open Approach | 20 |
| Surgery | 0DCM0ZZ | Extirpation of Matter from Descending Colon, Open Approach | 20 |
| Surgery | 0DQA0ZZ | Repair Jejunum, Open Approach | 20 |
| Surgery | 0D1N0Z4 | Bypass Sigmoid Colon to Cutaneous, Open Approach | 20 |
| Surgery | 0WUF0JZ | Supplement Abdominal Wall with Synthetic Substitute, Open Approach | 20 |
| Endoscopy | 0DCB8ZZ | Extirpation of Matter from Ileum, Via Natural or Artificial Opening Endoscopic | 20 |
| Endoscopy | 0DB48ZX | Excision of Esophagogastric Junction, Via Natural or Artificial Opening Endoscopic, Diagnostic | 20 |
| Surgery | 0DBL0ZZ | Excision of Transverse Colon, Open Approach | 15 |
| Surgery | 0DBN0ZZ | Excision of Sigmoid Colon, Open Approach | 15 |
| Surgery | 0DBU0ZZ | Excision of Omentum, Open Approach | 15 |
| Surgery | 0DC84ZZ | Extirpation of Matter from Small Intestine, Percutaneous Endoscopic Approach | 15 |
| Surgery | 0DQE0ZZ | Repair Large Intestine, Open Approach | 15 |
| Surgery | 0D1M0Z4 | Bypass Descending Colon to Cutaneous, Open Approach | 15 |
| Surgery | 0J980ZZ | Drainage of Abdomen Subcutaneous Tissue and Fascia, Open Approach | 15 |
| Surgery | 0DNW4ZZ | Release Peritoneum, Percutaneous Endoscopic Approach | 15 |
| Surgery | 0FN00ZZ | Release Liver, Open Approach | 15 |
| Surgery | 0WJF0ZZ | Inspection of Abdominal Wall, Open Approach | 15 |
| Surgery | 0WJP4ZZ | Inspection of Gastrointestinal Tract, Percutaneous Endoscopic Approach | 15 |
| Surgery | 0DH60UZ | Insertion of Feeding Device into Stomach, Open Approach | 15 |
| Surgery | 0D1B0Z4 | Bypass Ileum to Cutaneous, Open Approach | 15 |
| Surgery | 3E1M38Z | Irrigation of Peritoneal Cavity using Irrigat, Perc Approach Irrigation of Peritoneal Cavity using Irrigating Substance, Percutaneous Approach | 15 |
| Surgery | 0DU907Z | Supplement Duodenum with Autologous Tissue Substitute, Open Approach | 15 |
| Surgery | 0D9W0ZZ | Drainage of Peritoneum, Open Approach | 15 |
| Endoscopy | 0DCN7ZZ | Extirpation of Matter from Sigmoid Colon, Via Natural or Artificial Opening | 15 |
| Endoscopy | 0DBH8ZX | Excision of Cecum, Via Natural or Artificial Opening Endoscopic, Diagnostic | 15 |
| Endoscopy | 0WCP8ZZ | Extirpation of Matter from Gastrointestinal Tract, Via Natural or Artificial Opening Endoscopic | 15 |
| Endoscopy | 0CJY8ZZ | Inspection of Mouth and Throat, Via Natural or Artificial Opening Endoscopic | 15 |
| Endoscopy | 3E0G8GC | Introduction of Other Therapeutic Substance into Up GI, Endo Introduction of Other Therapeutic Substance into Upper GI, Via Natural or Artificial Opening Endoscopic | 15 |
| Surgery | 0DBA0ZZ | Excision of Jejunum, Open Approach | 10 |
| Surgery | 0DB84ZZ | Excision of Small Intestine, Percutaneous Endoscopic Approach | 10 |
| Surgery | 0DBF0ZZ | Excision of Right Large Intestine, Open Approach | 10 |
| Surgery | 0DBM0ZZ | Excision of Descending Colon, Open Approach | 10 |
| Surgery | 0DCC0ZZ | Extirpation of Matter from Ileocecal Valve, Open Approach | 10 |
| Surgery | 0DCF0ZZ | Extirpation of Matter from Right Large Intestine, Open Approach | 10 |
| Surgery | 0DCW0ZZ | Extirpation of Matter from Peritoneum, Open Approach | 10 |
| Surgery | 0DC40ZZ | Extirpation of Matter from Esophagogastric Junction, Open Approach | 10 |
| Surgery | 0DC94ZZ | Extirpation of Matter from Duodenum, Percutaneous Endoscopic Approach | 10 |
| Surgery | 0DCH4ZZ | Extirpation of Matter from Cecum, Percutaneous Endoscopic Approach | 10 |
| Surgery | 0DC54ZZ | Extirpation of Matter from Esophagus, Percutaneous Endoscopic Approach | 10 |
| Surgery | 0DQ20ZZ | Repair Middle Esophagus, Open Approach | 10 |
| Surgery | 0DQM0ZZ | Repair Descending Colon, Open Approach | 10 |
| Surgery | 0DQH0ZZ | Repair Cecum, Open Approach | 10 |
| Surgery | 0DQ70ZZ | Repair Stomach, Pylorus, Open Approach | 10 |
| Surgery | 0DQB0ZZ | Repair Ileum, Open Approach | 10 |
| Surgery | 0DQN0ZZ | Repair Sigmoid Colon, Open Approach | 10 |
| Surgery | 0JQ80ZZ | Repair Abdomen Subcutaneous Tissue and Fascia, Open Approach Repair Abdomen Subcutaneous Tissue and Fascia, Open Approach | 10 |
| Surgery | 0DNU4ZZ | Release Omentum, Percutaneous Endoscopic Approach | 10 |
| Surgery | 0W9F0ZZ | Drainage of Abdominal Wall, Open Approach | 10 |
| Surgery | 0D9E0ZZ | Drainage of Large Intestine, Open Approach | 10 |
| Surgery | 0W9G3ZZ | Drainage of Peritoneal Cavity, Percutaneous Approach | 10 |
| Surgery | 0DNH0ZZ | Release Cecum, Open Approach | 10 |
| Surgery | 0JD80ZZ | Extraction of Abdomen Subcutaneous Tissue and Fascia, Open Approach | 10 |
| Surgery | 0DJ60ZZ | Inspection of Stomach, Open Approach | 10 |
| Surgery | 0DJ64ZZ | Inspection of Stomach, Percutaneous Endoscopic Approach | 10 |
| Endoscopy | 0DCG8ZZ | Extirpation of Matter from Left Large Intestine, Via Natural or Artificial Opening Endoscopic | 10 |
| Endoscopy | 0DCE7ZZ | Extirpation of Matter from Large Intestine, Via Natural or Artificial Opening | 10 |
| Endoscopy | 0DBN8ZX | Excision of Sigmoid Colon, Via Natural or Artificial Opening Endoscopic, Diagnostic | 10 |
| Endoscopy | 0DBB8ZX | Excision of Ileum, Via Natural or Artificial Opening Endoscopic, Diagnostic | 10 |
| Endoscopy | 0DBL8ZX | Excision of Transverse Colon, Via Natural or Artificial Opening Endoscopic, Diagnostic | 10 |
| Endoscopy | 0DBK8ZX | Excision of Ascending Colon, Via Natural or Artificial Opening Endoscopic, Diagnostic | 10 |
| Endoscopy | 0DBM8ZZ | Excision of Descending Colon, Via Natural or Artificial Opening Endoscopic | 10 |
| Endoscopy | 0DP68YZ | Removal of Other Device from Stomach, Via Natural or Artificial Opening Endoscopic | 10 |
| Endoscopy | 0DC67ZZ | Extirpation of Matter from Stomach, Via Natural or Artificial Opening | 10 |
| Endoscopy | 0DC57ZZ | Extirpation of Matter from Esophagus, Via Natural or Artificial Opening | 10 |
| Endoscopy | 0DB18ZX | Excision of Upper Esophagus, Via Natural or Artificial Opening Endoscopic, Diagnostic | 10 |
| Endoscopy | 0D968ZZ | Drainage of Stomach, Via Natural or Artificial Opening Endoscopic | 10 |
| Endoscopy | 0D758ZZ | Dilation of Esophagus, Via Natural or Artificial Opening Endoscopic | 10 |
| Endoscopy | 0DP68DZ | Removal of Intraluminal Device from Stomach, Via Natural or Artificial Opening Endoscopic | 10 |
| Endoscopy | XW0G886 | Introduction of Mineral-based Topical Hemostatic Agent into Upper GI, Via Natural or Artificial Opening Endoscopic, New Technology Group 6 | 10 |
| Endoscopy | 0WJP7ZZ | Inspection of Gastrointestinal Tract, Via Natural or Artificial Opening Approach | 10 |
| Surgery | 0DB30ZZ | Excision of Lower Esophagus, Open Approach | 5 |
| Surgery | 0DBB4ZZ | Excision of Ileum, Percutaneous Endoscopic Approach | 5 |
| Surgery | 0DBU0ZX | Excision of Omentum, Open Approach, Diagnostic | 5 |
| Surgery | 0DTL0ZZ | Resection of Transverse Colon, Open Approach | 5 |
| Surgery | 0DB60Z3 | Excision of Stomach, Open Approach, Vertical | 5 |
| Surgery | 0DB20ZZ | Excision of Middle Esophagus, Open Approach | 5 |
| Surgery | 0DTB0ZZ | Resection of Ileum, Open Approach | 5 |
| Surgery | 0DBE0ZZ | Excision of Large Intestine, Open Approach | 5 |
| Surgery | 0DB64ZZ | Excision of Stomach, Percutaneous Endoscopic Approach | 5 |
| Surgery | 0DB40ZZ | Excision of Esophagogastric Junction, Open Approach | 5 |
| Surgery | 0DB64ZX | Excision of Stomach, Percutaneous Endoscopic Approach, Diagn Excision of Stomach, Percutaneous Endoscopic Approach, Diagnostic | 5 |
| Surgery | 0DTN0ZZ | Resection of Sigmoid Colon, Open Approach | 5 |
| Surgery | 0DBE8ZX | Excision of Large Intestine, Via Natural or Artificial Opening Endoscopic, Diagnostic | 5 |
| Surgery | 0DB80ZX | Excision of Small Intestine, Open Approach, Diagnostic | 5 |
| Surgery | 0DB10ZZ | Excision of Upper Esophagus, Open Approach | 5 |
| Surgery | 0DCV4ZZ | Extirpation of Matter from Mesentery, Percutaneous Endoscopic Approach | 5 |
| Surgery | 0DC34ZZ | Extirpation of Matter from Lower Esophagus, Percutaneous Endoscopic Approach | 5 |
| Surgery | 0DC50ZZ | Extirpation of Matter from Esophagus, Open Approach | 5 |
| Surgery | 0DCP4ZZ | Extirpation of Matter from Rectum, Percutaneous Endoscopic Approach | 5 |
| Surgery | 0DC30ZZ | Extirpation of Matter from Lower Esophagus, Open Approach | 5 |
| Surgery | 0DCG0ZZ | Extirpation of Matter from Left Large Intestine, Open Approach | 5 |
| Surgery | 0FC10ZZ | Extirpation of Matter from Right Lobe Liver, Open Approach | 5 |
| Surgery | 0WCPXZZ | Extirpation of Matter from Gastrointestinal Tract, External Approach | 5 |
| Surgery | 0DCQXZZ | Extirpation of Matter from Anus, External Approach | 5 |
| Surgery | 0DQW0ZZ | Repair Peritoneum, Open Approach | 5 |
| Surgery | 0DQB4ZZ | Repair Ileum, Percutaneous Endoscopic Approach | 5 |
| Surgery | 0DQ34ZZ | Repair Lower Esophagus, Percutaneous Endoscopic Approach | 5 |
| Surgery | 0DQK0ZZ | Repair Ascending Colon, Open Approach | 5 |
| Surgery | 0DQQ7ZZ | Repair Anus, Via Natural or Artificial Opening | 5 |
| Surgery | 0WUF07Z | Supplement Abdominal Wall with Autologous Tissue Substitute, Open Approach | 5 |
| Surgery | 0KXK0ZZ | Transfer Right Abdomen Muscle, Open Approach | 5 |
| Surgery | 0W3G0ZZ | Control Bleeding in Peritoneal Cavity, Open Approach | 5 |
| Surgery | 0WPF0YZ | Removal of Other Device from Abdominal Wall, Open Approach | 5 |
| Surgery | 0FT40ZZ | Resection of Gallbladder, Open Approach | 5 |
| Surgery | 0DP00UZ | Removal of Feeding Device from Upper Intestinal Tract, Open Approach | 5 |
| Surgery | 0W3P0ZZ | Control Bleeding in Gastrointestinal Tract, Open Approach | 5 |
| Surgery | 0KXL0ZZ | Transfer Left Abdomen Muscle, Open Approach | 5 |
| Surgery | 0D9W00Z | Drainage of Peritoneum with Drainage Device, Open Approach | 5 |
| Surgery | 0DNA4ZZ | Release Jejunum, Percutaneous Endoscopic Approach | 5 |
| Surgery | 0D1E0Z4 | Bypass Large Intestine to Cutaneous, Open Approach | 5 |
| Surgery | 07NP0ZZ | Release Spleen, Open Approach | 5 |
| Surgery | 0W9F00Z | Drainage of Abdominal Wall with Drainage Device, Open Approach | 5 |
| Surgery | 0D980ZZ | Drainage of Small Intestine, Open Approach | 5 |
| Surgery | 0WCJ0ZZ | Extirpation of Matter from Pelvic Cavity, Open Approach | 5 |
| Surgery | 0KXK0Z1 | Transfer Right Abdomen Muscle with Subcutaneous Tissue, Open Approach | 5 |
| Surgery | 0KBK0ZZ | Excision of Right Abdomen Muscle, Open Approach | 5 |
| Surgery | 0KBL0ZZ | Excision of Left Abdomen Muscle, Open Approach | 5 |
| Surgery | 0W9J30Z | Drainage of Pelvic Cavity with Drainage Device, Percutaneous Approach | 5 |
| Surgery | 0DHA0UZ | Insertion of Feeding Device into Jejunum, Open Approach | 5 |
| Surgery | 0W9G00Z | Drainage of Peritoneal Cavity with Drainage Device, Open Approach | 5 |
| Surgery | 0DUL07Z | Supplement Transverse Colon with Autologous Tissue Substitute, Open Approach | 5 |
| Surgery | 0D1L0Z4 | Bypass Transverse Colon to Cutaneous, Open Approach | 5 |
| Surgery | 0D870ZZ | Division of Stomach, Pylorus, Open Approach | 5 |
| Surgery | 0D190ZA | Bypass Duodenum to Jejunum, Open Approach | 5 |
| Surgery | 0DNB4ZZ | Release Ileum, Percutaneous Endoscopic Approach | 5 |
| Surgery | 0FN40ZZ | Release Gallbladder, Open Approach | 5 |
| Surgery | 0WCGXZZ | Extirpation of Matter from Peritoneal Cavity, External Approach | 5 |
| Surgery | 0W9F30Z | Drainage of Abdominal Wall with Drainage Device, Percutaneous Approach | 5 |
| Surgery | 0D9H0ZZ | Drainage of Cecum, Open Approach | 5 |
| Surgery | 0D1B4ZB | Bypass Ileum to Ileum, Percutaneous Endoscopic Approach | 5 |
| Surgery | 0DU60JZ | Supplement Stomach with Synthetic Substitute, Open Approach | 5 |
| Surgery | 0DJW4ZZ | Inspection of Peritoneum, Percutaneous Endoscopic Approach | 5 |
| Surgery | 0WUF4JZ | Supplement Abdominal Wall with Synth Sub, Perc Endo Approach Supplement Abdominal Wall with Synthetic Substitute, Percutaneous Endoscopic Approach | 5 |
| Surgery | 0BQT0ZZ | Repair Diaphragm, Open Approach | 5 |
| Surgery | 0W9G4ZZ | Drainage of Peritoneal Cavity, Percutaneous Endoscopic Approach | 5 |
| Surgery | 0DNL4ZZ | Release Transverse Colon, Percutaneous Endoscopic Approach | 5 |
| Surgery | 0DNW3ZZ | Release Peritoneum, Percutaneous Approach | 5 |
| Surgery | 0DNK0ZZ | Release Ascending Colon, Open Approach | 5 |
| Surgery | 0FT44ZZ | Resection of Gallbladder, Percutaneous Endoscopic Approach | 5 |
| Surgery | 0DNN0ZZ | Release Sigmoid Colon, Open Approach | 5 |
| Surgery | 0D9W30Z | Drainage of Peritoneum with Drainage Device, Percutaneous Approach | 5 |
| Surgery | 0DN90ZZ | Release Duodenum, Open Approach | 5 |
| Endoscopy | 0DDN8ZX | Extraction of Sigmoid Colon, Via Natural or Artificial Opening Endoscopic, Diagnostic | 5 |
| Endoscopy | 0DCQ7ZZ | Extirpation of Matter from Anus, Via Natural or Artificial Opening | 5 |
| Endoscopy | 0DCH7ZZ | Extirpation of Matter from Cecum, Via Natural or Artificial Opening | 5 |
| Endoscopy | 0DCL7ZZ | Extirpation of Matter from Transverse Colon, Via Natural or Artificial Opening | 5 |
| Endoscopy | 0DQB7ZZ | Repair Ileum, Via Natural or Artificial Opening | 5 |
| Endoscopy | 0DQP8ZZ | Repair Rectum, Via Natural or Artificial Opening Endoscopic | 5 |
| Endoscopy | 0DBM8ZX | Excision of Descending Colon, Via Natural or Artificial Opening Endoscopic, Diagnostic | 5 |
| Endoscopy | 0DBK8ZZ | Excision of Ascending Colon, Via Natural or Artificial Opening Endoscopic | 5 |
| Endoscopy | 0DBL8ZZ | Excision of Transverse Colon, Via Natural or Artificial Opening Endoscopic | 5 |
| Endoscopy | 0D9L8ZZ | Drainage of Transverse Colon, Via Natural or Artificial Opening Endoscopic | 5 |
| Endoscopy | 0DPQ8LZ | Removal of Artificial Sphincter from Anus, Via Natural or Artificial Opening Endoscopic | 5 |
| Endoscopy | 0DP58YZ | Removal of Other Device from Esophagus, Via Natural or Artificial Opening Endoscopic | 5 |
| Endoscopy | 0DD68ZX | Extraction of Stomach, Via Natural or Artificial Opening Endoscopic, Diagnostic | 5 |
| Endoscopy | 0DC87ZZ | Extirpation of Matter from Small Intestine, Via Natural or Artificial Opening | 5 |
| Endoscopy | 0D757ZZ | Dilation of Esophagus, Via Natural or Artificial Opening | 5 |
| Endoscopy | 0WCP7ZZ | Extirpation of Matter from Gastrointestinal Tract, Via Natural or Artificial Opening | 5 |
| Endoscopy | 0DC37ZZ | Extirpation of Matter from Lower Esophagus, Via Natural or Artificial Opening | 5 |
| Endoscopy | 0DB88ZX | Excision of Small Intestine, Via Natural or Artificial Opening Endoscopic, Diagnostic | 5 |
| Endoscopy | 0DQ98ZZ | Repair Duodenum, Via Natural or Artificial Opening Endoscopic | 5 |
| Endoscopy | 0DB58ZZ | Excision of Esophagus, Via Natural or Artificial Opening Endoscopic | 5 |
| Endoscopy | 0DBA8ZX | Excision of Jejunum, Via Natural or Artificial Opening Endoscopic, Diagnostic | 5 |
| Endoscopy | 0DB68ZZ | Excision of Stomach, Via Natural or Artificial Opening Endoscopic | 5 |
| Endoscopy | 0DQ28ZZ | Repair Middle Esophagus, Via Natural or Artificial Opening Endoscopic | 5 |
| Endoscopy | 0DQ48ZZ | Repair Esophagogastric Junction, Via Natural or Artificial Opening Endoscopic | 5 |
| Endoscopy | 0D738DZ | Dilation of Lower Esophagus with Intraluminal Device, Via Natural or Artificial Opening Endoscopic | 5 |
| Endoscopy | 0D728ZZ | Dilation of Middle Esophagus, Via Natural or Artificial Opening Endoscopic | 5 |
| Endoscopy | 0D758DZ | Dilation of Esophagus with Intraluminal Device, Via Natural or Artificial Opening Endoscopic | 5 |
| Endoscopy | 0DH68UZ | Insertion of Feeding Device into Stomach, Via Natural or Artificial Opening Endoscopic | 5 |
| Endoscopy | 0DH58DZ | Insertion of Intraluminal Device into Esophagus, Via Natural or Artificial Opening Endoscopic | 5 |
| Endoscopy | 3E1G88Z | Irrigation of Upper GI using Irrigating Substance, Via Natural or Artificial Opening Endoscopic | 5 |
| Endoscopy | 0DD78ZX | Extraction of Stomach, Pylorus, Via Natural or Artificial Opening Endoscopic, Diagnostic | 5 |
| Endoscopy | 0D568ZZ | Destruction of Stomach, Via Natural or Artificial Opening Endoscopic | 5 |
| Endoscopy | 0F798ZZ | Dilation of Common Bile Duct, Via Natural or Artificial Opening Endoscopic | 5 |
| Endoscopy | 0D778DZ | Dilation of Stomach, Pylorus with Intraluminal Device, Via Natural or Artificial Opening Endoscopic | 5 |
| Endoscopy | 0D998ZZ | Drainage of Duodenum, Via Natural or Artificial Opening Endoscopic | 5 |
| Endoscopy | 0D9680Z | Drainage of Stomach with Drainage Device, Via Natural or Artificial Opening Endoscopic | 5 |
| Endoscopy | 0D768ZZ | Dilation of Stomach, Via Natural or Artificial Opening Endoscopic | 5 |
| Endoscopy | 0D798DZ | Dilation of Duodenum with Intraluminal Device, Via Natural or Artificial Opening Endoscopic | 5 |
| Endoscopy | 0DH57DZ | Insertion of Intraluminal Device into Esophagus, Via Opening Insertion of Intraluminal Device into Esophagus, Via Natural or Artificial Opening | 5 |
|  |  |  |  |

Table S3. Univariable regression analysis assessing factors associated with the need for surgical intervention among patients hospitalized with self-harm–associated gastrointestinal foreign bodies.

| Variable | Odds ratio (95% CI) | *P-*value |
| --- | --- | --- |
| Object location |  |  |
| Stomach | Ref. | - |
| Esophagus | 0.80 (0.49-1.33) | 0.397 |
| Small bowel | 2.52 (1.93-3.29) | <0.001 |
| Colon | 1.22 (0.89-1.69) | 0.219 |
| Rectum or anus | 1.21 (0.69-2.14) | 0.503 |
| Sharp object | 1.21 (0.95-1.55) | 0.116 |
| Female sex | 0.77 (0.60-0.98) | 0.037 |
| Incarceration | 0.76 (0.57-1.01) | 0.058 |
| Cluster B personality disorder | 0.70 (0.55-0.89) | 0.004 |
| Teaching hospital | 0.66 (0.50-0.88) | 0.005 |
| Age 50+ | 1.16 (0.81-1.65) | 0.414 |
| Charlson Comorbidity Index of 3 or more | 0.53 (0.27-1.06) | 0.073 |
| Mental health diagnosis | 0.89 (0.63-1.26) | 0.503 |

Table S4. Univariable regression analysis assessing factors associated with the need for surgical intervention after initial endoscopic attempt among patients hospitalized with self-harm–associated gastrointestinal foreign bodies.

| Variable | Odds ratio (95% CI) | *P-*value |
| --- | --- | --- |
| Object location |  |  |
| Stomach | Ref. | - |
| Esophagus | 0.97 (0.56-1.68) | 0.906 |
| Small bowel | 2.44 (1.67-3.58) | <0.001 |
| Colon | 1.49 (0.98-2.26) | 0.062 |
| Rectum or anus | 1.16 (0.57-2.38) | 0.680 |
| Sharp object | 1.13 (0.81-1.57) | 0.476 |
| Female sex | 0.87 (0.65-1.18) | 0.370 |
| Incarceration | 1.02 (0.72-1.45) | 0.897 |
| Cluster B personality disorder | 0.75 (0.56-1.01) | 0.058 |
| Teaching hospital | 0.76 (0.52-1.10) | 0.145 |
| Age 50+ | 0.99 (0.61-1.61) | 0.971 |
| Charlson Comorbidity Index of 3 or more | 0.38 (0.14-1.04) | 0.060 |
| Mental health diagnosis | 0.96 (0.60-1.53) | 0.853 |

Table S5. Multivariable regression analysis assessing factors associated with the need for surgical intervention among patients hospitalized with self-harm–associated gastrointestinal foreign bodies.

| Variable | Odds ratio (95% CI) | *P-*value |
| --- | --- | --- |
| Object location |  |  |
| Stomach | Ref. | - |
| Esophagus | 0.80 (0.48-1.34) | 0.401 |
| Small bowel | 2.50 (1.91-3.28) | <0.001 |
| Colon | 1.16 (0.84-1.61) | 0.374 |
| Rectum or anus | 0.99 (0.56-1.77) | 0.981 |
| Sharp object | 1.14 (0.87-1.49) | 0.330 |
| Female sex | 0.78 (0.59-1.03) | 0.079 |
| Incarceration | 0.58 (0.42-0.81) | 0.001 |
| Cluster B personality disorder | 0.78 (0.60-1.01) | 0.060 |
| Teaching hospital | 0.66 (0.48-0.89) | 0.006 |
| Age 50+ | 1.22 (0.83-1.78) | 0.307 |
| Charlson Comorbidity Index of 3 or more | 0.57 (0.27-1.19) | 0.307 |
| Mental health diagnosis | 0.84 (0.59-1.20) | 0.343 |

Table S6. Multivariable regression analysis assessing factors associated with the need for surgical intervention after initial endoscopic attempt among patients hospitalized with self-harm–associated gastrointestinal foreign bodies.

| Variable | Odds ratio (95% CI) | *P-*value |
| --- | --- | --- |
| Object location |  |  |
| Stomach | Ref. | - |
| Esophagus | 0.97 (0.56-1.69) | 0.920 |
| Small bowel | 2.47 (1.68-3.63) | <0.001 |
| Colon | 1.43 (0.94-2.18) | 0.092 |
| Rectum or anus | 1.02 (0.48-2.13) | 0.966 |
| Sharp object | 1.00 (0.69-1.45) | 0.998 |
| Female sex | 0.90 (0.63-1.28) | 0.553 |
| Incarceration | 0.87 (0.58-1.30) | 0.495 |
| Cluster B personality disorder | 0.78 (0.56-1.08) | 0.135 |
| Teaching hospital | 0.77 (0.53-1.14) | 0.189 |
| Age 50+ | 1.14 (0.70-1.86) | 0.587 |
| Charlson Comorbidity Index of 3 or more | 0.42 (0.15-1.16) | 0.094 |
| Mental health diagnosis | 0.99 (0.60-1.63) | 0.973 |

Table S7. Characteristics of hospitalizations for gastrointestinal foreign bodies in patients with self-harm intention, stratified by incarceration status

|  | Non-incarcerated | Incarcerated | *P*-value |
| --- | --- | --- | --- |
| Age, years % (n = 16,150) |  |  |  |
| 18-30 | 43.4 | 40.7 | 0.191 |
| 31-49 | 46.6 | 55.6 | <0.001 |
| 50+ | 10.1 | 3.3 | <0.001 |
| Female, % (n = 16,140) | 43.1 | 6.5 | <0.001 |
| Race, % (n = 13,735) |  |  |  |
| White | 66.4 | 44.6 | <0.001 |
| Black | 18.7 | 27.7 | <0.001 |
| Hispanic | 10.2 | 20.8 | <0.001 |
| Asian or pacific islander | 1.1 | 1.1 | 0.970 |
| Other | 3.6 | 5.9 | 0.019 |
| United States hospital region, % (n = 14,015) |  |  |  |
| Northeast | 17.9 | 12.4 | 0.020 |
| Midwest | 24.6 | 11.6 | <0.001 |
| South | 37.3 | 30.1 | 0.031 |
| West | 20.2 | 45.9 | <0.001 |
| Teaching hospital, % (n = 16,150) | 83.7 | 86.8 | 0.089 |
| Charlson Comorbidity Index, % (n = 16,150) |  |  |  |
| 0 | 57.1 | 64.3 | <0.001 |
| 1 | 30.1 | 27.8 | 0.076 |
| 2 | 8.7 | 5.9 | 0.016 |
| 3+ | 4.2 | 3.1 | 0.160 |
| Major depressive disorder, % (n = 16,150) | 36.5 | 31.0 | 0.009 |
| Generalized anxiety disorder, % (n = 16,150) | 33.4 | 23.7 | <0.001 |
| Post-traumatic stress disorder, % (n = 16,150) | 29.8 | 17.0 | <0.001 |
| Bipolar disorder, % (n = 16,150) | 35.9 | 31.8 | 0.047 |
| Schizophrenia, % (n = 16,150) | 41.2 | 33.0 | <0.001 |
| Cluster B personality disorder, % (n = 16,150) | 39.1 | 22.9 | <0.001 |
| Concurrent genitourinary foreign body, % (n = 16,150) | 1.8 | 5.3 | <0.001 |
| Sharp object, % (n = 16,150) | 18.2 | 30.0 | <0.001 |
| Object location, % (n = 16,150) |  |  |  |
| Esophagus | 6.9 | 5.6 | 0.258 |
| Stomach | 43.9 | 43.2 | 0.722 |
| Small bowel | 14.5 | 16.7 | 0.132 |
| Colon | 13.2 | 15.6 | 0.109 |
| Rectum or anus | 3.8 | 3.1 | 0.347 |
| Not specified | 17.8 | 15.9 | 0.241 |

Table S8. Multivariable logistic regression assessing factors associated with the need for surgical intervention among non-incarcerated patients hospitalized with self-harm–associated gastrointestinal foreign bodies.

| Variable | Odds ratio (95% CI) | *P*-value |
| --- | --- | --- |
| Object location |  |  |
| Stomach | Ref. | - |
| Esophagus | 0.76 (0.43-1.34) | 0.337 |
| Small bowel | 2.70 (1.99-3.68) | <0.001 |
| Colon | 1.11 (0.77-1.59) | 0.582 |
| Rectum or anus | 1.08 (0.59-2.01) | 0.796 |
| Sharp object | 1.22 (0.90-1.65) | 0.204 |
| Female sex | 0.78 (0.58-1.04) | 0.096 |
| Cluster B personality disorder | 0.77 (0.58-1.03) | 0.064 |
| Teaching hospital | 0.72 (0.52-1.00) | 0.048 |

Table S9. Multivariable logistic regression assessing factors associated with the need for surgical intervention after initial endoscopic attempt among non-incarcerated patients hospitalized with self-harm–associated gastrointestinal foreign bodies.

| Variable | Odds ratio (95% CI) | *P*-value |
| --- | --- | --- |
| Object location |  |  |
| Stomach | Ref. | - |
| Esophagus | 0.91 (0.48-1.71) | 0.769 |
| Small bowel | 2.66 (1.72-4.10) | <0.001 |
| Colon | 1.34 (0.83-2.18) | 0.232 |
| Rectum or anus | 1.03 (0.45-2.31) | 0.952 |
| Sharp object | 1.07 (0.69-1.64) | 0.769 |
| Female sex | 0.90 (0.62-1.30) | 0.578 |
| Cluster B personality disorder | 0.77 (0.54-1.11) | 0.167 |
| Teaching hospital | 0.81 (0.53-1.24) | 0.332 |

Table S10. Multivariable logistic regression assessing factors associated with the need for surgical intervention among incarcerated patients hospitalized with self-harm–associated gastrointestinal foreign bodies.

| Variable | Odds ratio (95% CI) | *P*-value |
| --- | --- | --- |
| Object location |  |  |
| Stomach | Ref. | - |
| Esophagus | 1.06 (0.33-3.36) | 0.922 |
| Small bowel | 1.91 (1.02-3.60) | 0.045 |
| Colon | 1.30 (0.64-2.65) | 0.473 |
| Rectum or anus | 0.44 (0.06-3.11) | 0.408 |
| Sharp object | 0.86 (0.47-1.59) | 0.629 |
| Female sex | 1.00 (0.37-2.73) | 0.998 |
| Cluster B personality disorder | 0.86 (0.49-1.63) | 0.636 |
| Teaching hospital | 0.43 (0.21-0.87) | 0.019 |

Table S11. Multivariable logistic regression assessing factors associated with the need for surgical intervention after initial endoscopic attempt among incarcerated patients hospitalized with self-harm–associated gastrointestinal foreign bodies.

| Variable | Odds ratio (95% CI) | *P*-value |
| --- | --- | --- |
| Object location |  |  |
| Stomach | Ref. | - |
| Esophagus | 1.17 (0.36-3.76) | 0.791 |
| Small bowel | 1.90 (0.80-4.49) | 0.145 |
| Colon | 1.64 (0.67-4.01) | 0.278 |
| Rectum or anus | 0.70 (0.10-5.17) | 0.730 |
| Sharp object | 0.80 (0.40-1.61) | 0.532 |
| Female sex | 1.13 (0.37-3.49) | 0.827 |
| Cluster B personality disorder | 0.88 (0.43-1.78) | 0.717 |
| Teaching hospital | 0.62 (0.25-1.49) | 0.284 |

Table S12. Need for ICU-level care by management pathway among patients hospitalized with self-harm–associated gastrointestinal foreign bodies.

| Outcome | No intervention (n = 4,270) | Endoscopy only  (n = 9,880) | Endoscopy followed by surgery  (n = 1,120) | Surgery only  (n = 780) | Surgery followed by endoscopy  (n = 100) | *P-*value |
| --- | --- | --- | --- | --- | --- | --- |
| ICU-level care | 15 (0.4%) | 375 (3.8%) | 55 (4.9%) | 10 (1.3%) | 5 (5.0%) | <0.001 |

Note: ICU-level care was defined as the presence of any of the following: positive pressure ventilation for greater than 24 consecutive hours, mechanical ventilation for greater than 24 consecutive hours, vasopressor use, or cardiopulmonary resuscitation.

Table S13. Multivariable logistic regression assessing factors associated with no intervention being performed among patients hospitalized with self-harm–associated gastrointestinal foreign bodies.

| Variable | Odds ratio (95% CI) | *P*-value |
| --- | --- | --- |
| Object location |  |  |
| Stomach | Ref. | - |
| Esophagus | 0.62 (0.39-1.00) | 0.049 |
| Small bowel | 2.87 (2.26-3.63) | <0.001 |
| Colon | 2.82 (2.21-3.60) | <0.001 |
| Rectum or anus | 1.08 (0.64-1.81) | 0.767 |
| Sharp object | 1.01 (0.81-1.27) | 0.917 |
| Female sex | 0.83 (0.66-1.04) | 0.099 |
| Incarceration | 1.55 (1.24-1.93) | <0.001 |
| Cluster B personality disorder | 0.85 (0.69-1.06) | 0.152 |
| Teaching hospital | 1.05 (0.77-1.38) | 0.734 |

Table S14. Need for surgical intervention and surgical intervention after endoscopy among patients hospitalized with self-harm–associated gastrointestinal foreign bodies, stratified by year

|  | 2016 | 2017 | 2018 | 2019 | 2020 | 2021 | 2022 | 2023 | *P*-value |
| --- | --- | --- | --- | --- | --- | --- | --- | --- | --- |
| Surgical intervention, % | 11.4 | 11.7 | 12.8 | 14.9 | 12.0 | 15.3 | 9.8 | 10.8 | 0.370 |
| Surgical intervention after endoscopy, % | 9.5 | 9.2 | 10.3 | 9.8 | 10.1 | 12.3 | 9.2 | 10.7 | 0.959 |
